# Radioprotection of deinococcal exopolysaccharide BRD125 by regenerating hematopoietic stem cells

**DOI:** 10.3389/fonc.2022.898185

**Published:** 2022-09-26

**Authors:** Hae Ran Park, Ji Hee Lee, Hyun Jung Ji, Sangyong Lim, Ki Bum Ahn, Ho Seong Seo

**Affiliations:** ^1^ Research Division for Radiation Science, Korea Atomic Energy Research Institute, Jeongeup, South Korea; ^2^ Division of Pathogen Resource Management, Center for Public Vaccine Development Support, National Institute of Infectious Diseases, National Institute of Health (NIH), Korea Disease Control and Prevention Agency, Cheongju, South Korea; ^3^ Department of Oral Microbiology and Immunology, DRI, and BK21 Plus Program, School of Dentistry, Seoul National University, Seoul, South Korea; ^4^ Department of Radiation Science, University of Science and Technology, Daejeon, South Korea

**Keywords:** *Deinococcus*, exopolysaccharide, DeinoPol, hematopoietic stem cell (HSC), radiation, radioprotectant, mitigator, radiotherapy

## Abstract

There is a substantial need for the development of biomaterials for protecting hematopoietic stem cells and enhancing hematopoiesis after radiation damage. Bacterial exopolysaccharide (EPS) has been shown to be very attractive to researchers as a radioprotectant owing to its high antioxidant, anti-cancer, and limited adverse effects. In the present study, we isolated EPS from a novel strain, *Deinococcus radiodurans* BRD125, which produces EPS in high abundance, and investigated its applicability as a radioprotective biomaterial. We found that EPS isolated from EPS-rich *D. radiodurans* BRD125 (DeinoPol-BRD125) had an excellent free-radical scavenging effect and reduced irradiation-induced apoptosis. In addition, bone-marrow and spleen-cell apoptosis in irradiated mice were significantly reduced by DeinoPol-BRD125 administration. DeinoPol-BRD125 enhanced the expression of hematopoiesis-related cytokines such as GM-CSF, G-GSF, M-CSF, and SCF, thereby enhancing hematopoietic stem cells protection and regeneration. Taken together, our findings are the first to report the immunological mechanism of a novel radioprotectant, DeinoPol-BRD125, which might constitute an ideal radioprotective and radiation mitigating agent as a supplement drug during radiotherapy.

## Introduction

Although ionizing radiation (IR) is known to be harmful, the use of radiation is increasing in many as of industry and medicine (1–3). Because IR-induced cellular damage comprises a very diverse and complex sequence of molecular events, it is very difficult to protect and prevent (4). IR-induced damage ultimately results from excessive production of multiple free radicals induced by radiolysis (4–6). Therefore, many researchers have suggested that the use of scavengers to reduce free radical levels could be a plausible strategy to prevent radiation-induced disruption of hematopoiesis (7, 8). Although many antioxidants that can scavenge free radicals have been studied as candidate radioprotectants, most of them are ineffective (9–11). This may mean that the nature of radiation-induced damage is very diverse owing to secondary radical species, and the treatment to control them must be applied differently depending on the type of radiation-induced damage (12–15). Accordingly, radiation mitigating or protecting agents have been developed to prevent these cascades or intervene to prevent the perpetuation of damage and thus reduce the expression of toxicity.

Amifostine (2-[(3-aminopropyl)amino]ethane-thiol dihydrogen phosphate ester; WR-2721), which provides free thiols to adjacent normal tissues (16–18), is the only drug that has been approved by the FDA to minimize RT-induced damage. However, its clinical use is rather limited owing to severe toxicities such as nausea, emesis, and hypotension (19, 20). Palifermin is a recombinant human keratinocyte growth factor (KGF) that acts specifically on the differentiation, proliferation, and survival of KGF receptor-expressing epithelial cells (21–23). It has been approved by the FDA to reduce the incidence and duration of severe oral mucositis by enhancing Th2-type cytokines and reducing DNA strand break-mediated apoptosis (18, 24, 25). Long term clinical studies have generally reported it to be well tolerated with mild adverse events, and without the development of secondary malignancies.

Acute radiation syndrome (ARS) is a disease caused by middle- and high-dose IR, which develops into various subtypes of ARS, including hematopoietic, gastrointestinal, and cardiovascular syndromes, as it depletes immature parenchymal stem cells in specific tissues (11, 26–28). Among them, hematopoietic ARS (bone marrow syndrome, radiation-acquired aplastic anemia) is the most frequently occurring IR-induced injury. Hematopoietic ARS severely depletes multi-lineage blood cells in the bone marrow (BM), in the crypts of the small intestine, and in the basal layer of the skin (29, 30). The primary cause of death is the destruction of the BM, resulting in life-threatening infections and hemorrhage (27). Therefore, preventing the destruction of bone marrow cells (BMCs) is the primary target of drugs intended to improve or prevent hematopoietic ARS.

Polysaccharides extracted from natural sources such as medicinal herbs, mushrooms, or algae are attracting attention owing to their diverse pharmacological applications and low toxicity (31–33). Interestingly, polysaccharides have long been studied for their ability to react with the reactive oxygen species (ROS) produced by the ionization of water and other molecules as well as to enhance immune function (34, 35). Fucoidan and carrageenan from algae have been extensively investigated for their radioprotective effects (31, 34, 35). An acidic polysaccharide isolated from *Panax ginseng* has been demonstrated to have radioprotective, anti-tumor, and immuno-modulatory effects (36–41).

Exopolysaccharides (EPSs) are carbohydrate polymers classified as homo- or hetero-polysaccharides that have attracted attention in recent years owing to their interesting functional properties such as increasing longevity, limiting radiation damage, and tumor growth inhibition (42–44). Various EPSs from bacteria, fungi, and mushrooms have been studied. Furthermore, it has been shown that modified EPS obtained by acetylation, phosphorylation, or carboxymethylation of EPS exhibited greater efficacy as antioxidant and antitumor materials (45–47). In a previous study, we reported that EPS isolated from *Deinococcus radiodurans* R1 strain (DeinoPol) demonstrates highly protective effects on human keratinocytes in response to stress-induced apoptosis by effectively scavenging ROS (48). *D. radiodurans* is a non-pathogenic bacterium that is extremely resistant to extracellular stresses such as ionizing radiation, desiccation, UV radiation, and oxidizing agents (49, 50).

In the present study, we isolated a novel *D. radiodurans* BRD125 strain from soil obtained from the Baegnokdam of Mt. Halla in the Republic of Korea and then purified EPSs as high-value biomolecules from this bacteria. Moreover, we investigated whether this EPS (DeinoPol-BRD125) protects cells against IR and possesses regenerative properties. We propose DeinoPol-BRD125 as both a radioprotectant and a radiomitigator in mice.

## Materials and methods

### Isolation and identification of bacterial strain, *D. radiodurans* BRD125

Strain BRD125 was isolated from a soil sample obtained from Baegnokdam lake of Mt. Halla in the Republic of Korea. The soil sample was exposed to 5 kGy at room temperature using a ^60^Co-γ irradiator (AECL, IR-79; MDS Nordion International Co., Ltd.) at the Advanced Radiation Technology Institute in Republic of Korea. R2A liquid medium was added to the irradiated soil sample, which was then incubated for 2 hours at 30°C for enrichment. The sample was serially diluted with saline solution and spread on R2A agar (MB Cell; Seoul, Korea). After plating, plates were incubated at 30°C for 3 days. The isolate was transferred onto R2A fresh plate and incubated at 30°C for 2 days.

For identification of strain BRD125, genomic DNA was extracted using a G-spin™ Genomic DNA Extraction Kit (iNtRON; Seongnam-Si, Korea) following the manufacturer’s instructions. The 16S rRNA gene was amplified using the universal primers 27F (5′- AGAGTTTGATCMTGGCTCAG-3′) and 1492R (5′- TACGGYTACCTTGTTACGACTT-3′) as described previously (51). The PCR product was sequenced by Macrogen Co., Ltd. (Seoul, Korea). The 16S rRNA gene sequence (1,390 bp) of strain BRD125 was obtained and the similarity was confirmed using the EzBioCloud server (www.ezbiocloud.net; Seoul, Korea). BRD125 showed the highest 16S rRNA gene sequence similarity to *Deinococcus radiodurans* with a similarity of 99.7%.

### Whole-genome sequencing, genome assembly, and annotation

*D. radiodurans* BRD125 strain was sequenced using PacBio single-molecule sequencers (Pacific Biosciences; Menlo Park, CA, USA) by Macrogen Inc. (Seoul, South Korea). *De novo* assembly was implemented using the hierarchical genome assembly process version 3 (HGAP3). The genome annotations were performed using the PROKKA pipeline (v1.13), and gene functions were identified using eggNOG (52, 53). The GenBank accession number for the genomic sequence of the *D. radiodurans* BRD125 is SAMN15246708. Comparative genomic analysis was performed by analyzing ANI for the nucleotide level comparison with *D. radiodurans* R1 retrieved from NCBI GenBank database (54). Genome-wide visualization of coding sequence identity between BRD125 and R1 was constructed using BRIG.

### EPS purification from *D. radiodurans* BRD125

Microbial EPS was purified as described previously (48, 55). *D. radiodurans* BRD125 strain was cultured in 4 L of 2×TGY broth at 30°C with shaking at 900 rpm. After 48 h of incubation, the culture was mixed with 0.1% deoxycholate to lyse the bacterial cell wall and heated at 100°C for 10 min to inactivate the bacteria and enzymes. Subsequently, the cells were removed by centrifugation (10,000 × *g*, 30 min, 4°C). The supernatant was concentrated and dialyzed using a Minimate tangential flow filtration system with 30 K Minimate capsule (Pall Life Sciences; Port Washington, NY, USA). The concentrated supernatant (approximately 40 mL) was precipitated with 160 mL of 95% ethanol (Daejungchem; Seoul, Korea) at 4°C for 12 h, and the precipitate was collected by centrifugation (5000 × *g*, 10 min, 4°C) to yield the crude polysaccharide solution. The proteins in the crude polysaccharide were removed by the Sevage method as described previously (56, 57). Briefly, the precipitate was dissolved in 10 mL of distilled water and mixed with 30 mL chloroform:*n*-butanol (4:1 v/v). After vigorous shaking for 5 min, the mixture was allowed to stand for 15 min, and the aqueous phase was collected and precipitated with 80% ethanol. After filtration with a 0.22-μm Millex-GP syringe filter unit (Merck Millipore; Burlington, MA, USA), the final product was lyophilized.

### Cell lines and X-ray irradiation

Chinese Hamster Ovary (CHO)-K1 cell lines were purchased from American Type Culture Collection (ATCC; Manassas, VA, USA). Cells were cultured in Dulbecco’s modified Eagle’s medium (Life Technologies, Inc., Carlsbad, CA, USA) supplemented with 10% heat-inactivated fetal bovine serum (Life Technologies, Inc) and antibiotics (100 U/ml penicillin and 0.1 mg/ml streptomycin; Life Technologies, Inc) at 37°C in a standard humidified 5% CO_2_ atmosphere. Cells were irradiated with 160 kV, 1 mA X-ray at a dose rate of 0.3 Gy/min using a cabinet X-Radiator™ system (Faxitron; Wheeling, IL, USA).

### DPPH radical scavenging activity

The scavenging activity of EPS against 1, 1-diphenyl-2-picrylhydraxyl (DPPH) radical was evaluated spectrophotometrically using a previously described method (58) with a modification. Briefly, 990 μl of DPPH methanol solution (0.15 mM; Sigma-Aldrich; St. Louis, MO, USA) was added to a tube containing 10 μl of EPS. After incubation in the dark for 30 min, the absorbance was measured at 517 nm using an Epoch2 Microplate Spectrophotometer (BioTek; Winooski, VT, USA). The scavenging activity was determined by the following equation:


$$DPPH\;radical\;scavenging\;activity\;(\%)=\;\frac{A_{control}\;–\;A_{sample}}{A_{control}\;–\;A_{blank}}\;\times \;100$$

Where A_control_ is the absorbance of mixture without sample, A_sample_ is the absorbance of reaction mixture with sample, and A_blank_ is the absorbance of methanol.

### Measurement of intracellular ROS

Intracellular ROS levels were examined using chloromethyl-2, 7-dichlorofluorescin diacetate (DCFH-DA; Sigma-Aldrich) *via* MACSQuant Analyzer flow cytometry (North Rhine-Westphalia, Germany). Briefly, CHO-K1 cells were cultured with or without 50 μg/ml BRD125-EPS. After 24 hours, the cells were washed with phosphate-buffered saline (PBS), followed by incubation with 20 μM DCHF-DA at 37°C in the dark for 1 h. The cells were harvested and immediately exposed to 4 Gy of X-ray (160 kV, 1 mA). After irradiation, the cells were analyzed by flow cytometry. At least 10,000 events were analyzed.

### Colony forming assay (Clonogenic assay)

Clonogenic assay was performed as described previously (59). CHO-K1 cells were seeded into six-well plates with or without 50 μg/ml DeinoPol-BRD125. After 2 hours, the cells were exposed to various doses of X-rays and subsequently incubated for 7 days. The medium of all cultures was replaced every 3 days. The colonies were fixed with 70% methanol and stained with 0.5% crystal violet. Colonies containing 50 cells or more were counted as clonogenic cells. Surviving fraction (SF) and plating efficacy (PF) were calculated using the equations:


$$SF\;=\;\frac{PF\;of\;testgroup}{PF\;of\;control\;group}\;$$

and


$$PF\;=\;\frac{No.\;of\;colonies\;}{No.\;of\;seeding\;cells}\;\times \;100$$

The reported survival fraction values are the mean of six replicates from three independent experiments.

### *In vitro* apoptosis analysis

BMCs and spleen cells (3×10^6^) were plated on 12-well culture plates with or without 50 μg/ml DeinoPol-BRD125 for 2 h followed by 2 Gy X-ray irradiation. Apoptotic cells were counted by staining with propidium iodide (PI) (Sigma-Aldrich). Briefly, cultured cells were washed once with PBS and fixed with 70% ethanol at -20°C overnight. The fixed cells were resuspended in 1 ml of PI buffer [0.5 mg/ml RNaseA (Sigma-Aldrich) and 0.1 mg/ml PI (Sigma-Aldrich) in PBS]. After incubation for 30 minutes in the dark, the PI of individual nuclei were analyzed by MACSQuant flow cytometry (Miltenyi Biotech; Bergisch Gladbach, Germany).

### Bromodeoxyuridine proliferation assay of spleen lymphocytes *in vitro*

Proliferation of spleen lymphocytes was detected using the colorimetric bromodeoxyuridine (BrdU) proliferation assay kit (Cell Signaling), according to the manufacturer’s instructions. Spleen cells (3×10^5^) were incubated on 96-well culture plates without or with 50 μg/ml DeinoPol-BRD125 for 2 h followed by 1 Gy X-ray irradiation. At 1 or 3 days after irradiation, the spleen lymphocytes were treated with BrdU for 12 h and BrdU incorporated into cellular DNA was detected using a BrdU mAb according to the manufacturer’s instructions.

### Mouse experiment and irradiation

The animal housing conditions, which were designed for specific pathogen-free animals, and the animal experimental design were approved by the Committee on the Use and Care of Animals at KAERI and implemented ethically according to the standards accepted by the National Institutes of Health. The ventilated housing cage (Orient Bio Inc., Seoul, Korea) was maintained in an animal biological safety level 2 facility at 22–23°C on a 12 h:12 h light:dark cycle. Six-week-old female C57BL/6 mice were purchased from Orient Bio Inc (Seongnam, South Korea).

Mouse irradiation was conducted at room temperature using a Gammacell 40 Exactor (Nordion International Inc. Ottawa, Canada). The mice were irradiated at 6.5 Gy for the experiment on endogenous spleen colony formation assay and at 4 Gy for other experiments. The dose rate was 0.9 Gy/min of ^137^Cs γ-ray. DeinoPol-BRD125 was dissolved in PBS and then intraperitoneally injected 36 h and 12 h before irradiation, and 30 min, 24 h, and 48 h after irradiation at a dose of 50 mg/body weight (BW).

### Mice cell preparation

The spleen and BM were isolated aseptically from the C57BL/6 mice. Spleen cells were prepared by filtering through a cell strainer (70 µm; SPL Life Sciences, Pocheon, Korea) and BMCs was collected by flushing from mice femurs with PBS. Red blood cells (RBCs) in spleen cells and BMCs were removed using RBC lysis buffer (Sigma-Aldrich). All cell suspensions were resuspended in RPMI1640 (Gibco; Grand Island, NY, USA) supplemented with 10% fetal bovine serum.

### DNA fragmentation analysis

BMCs and spleen cells that were harvested at 4 h after irradiation were lysed for 30 min with 400 μl of lysis buffer (10 mM Tris-HCl, pH 8.0, 20 mM EDTA, 0.5% Trion X-100) on ice. After centrifugation, the supernatants were extracted twice with equal volumes of equilibrated phenol:chloroform solution (v:v=4:1). After additions of 0.1 volumes of 5 M sodium chloride and 2.5 volumes of ethanol, samples were stored at -20°C overnight to precipitate chromosomal DNA. After washing with ice-cold 70% ethanol, the pellets were dissolved in Tris/EDTA buffer (10 mM Tris-HCl, pH 8.0, 1 mM EDTA) containing 30 μg/ml RNase and incubated at 37°C for 3 hours. Extracted DNA were visualized in 2% agarose gel containing 0.1% ethidium bromide (Sigma-Aldrich).

### Endogenous spleen colony-forming units assay

An endogenous CFU assay was performed to confirm the effect of DeinoPol-BRD125 on hematopoietic ability (60, 61). The C57BL/6 mice were sacrificed 9 days after irradiation with 6.5 Gy. The spleens were removed and then fixed in Bouin’s solution (Sigma-Aldrich). After 1 day, the colonies on the surfaces of the spleens were counted.

### Analysis of peripheral blood cells

Whole blood was collected from the retro-orbital veins using heparin coated-capillary tubes and then collected in VACUETTE K3EDTA tubes. The absolute number of monocytes, lymphocytes, white blood cells (WBCs), RBCs, neutrophils, and platelets in blood was counted using an automatic blood analyzer (Hemavet 950; CDC Technologies Inc.; Dayton, OH, USA).

### Bone marrow cell transplantation assay

The effects of DeinoPol-BRD125 on hematopoiesis were studied *in vivo* using BM cells transplantation assay. Donor mice were intraperitoneally injected with PBS or DeinoPol-BRD125 at 36 h and 12 h before irradiation with 4 Gy, and 30 min, 24 h, and 48 h after irradiation. On day 7 after irradiation, BM cells from donor mice were harvested and 2×10^6^ BM cells were injected into the tail veins of recipient mice, which were lethally irradiated (8 Gy) 6 h earlier. Two and four weeks after transplantation, cells in peripheral blood were counted using an automatic blood analyzer (Hemavet 950; Drew Scientific; Miami Lakes, Florida, USA), and cell populations in the spleen were analyzed by MACSQuant flow cytometry (Miltenyi Biotech).

### Analysis of immune cells in the spleen

Splenocytes were incubated with anti-mouse CD16/CD32 and then stained with all antibodies and 7-aminoactinomycin D (7-AAD) for 30 min at 4°C. After staining, the cells were washed three times and were counted using MACSQuant flow cytometry (Miltenyi Biotech). The following antibodies were used: Purified rat anti-mouse CD16/CD32, BV421-conjugated anti-mouse F4/80, APC-conjugated anti-mouse CD45R/B220, V500 simian-conjugated anti-mouse CD3e, FITC-conjugated anti-mouse NK1.1 were purchased from BD Biosciences (San Diego, CA, USA). PE-Cy7-conjugated anti-mouse MHC-II and PE-conjugated CD11c were purchased from eBioscience. PE-Texas Red-conjugated anti-mouse CD11b were purchased from Thermo Fisher Scientific (Waltham, MA, USA).

### Quantitative real-time polymerase chain reaction

Three days after irradiation, BMCs and spleen cells were collected and total RNA was isolated using TRIzol Reagent (Thermo Fisher Scientific; Wiltham, MA, USA) according to the manufacturer’s protocol. Reverse transcription from three micrograms of total RNA was performed using random primers, dNTP mixture, and MMLV reverse transcriptase (Promega; Madison WI, USA). Quantitative real-time PCR was performed using a StepOne Real-Time PCR (Applied Biosystems; CA, USA) with SYBR Green reagent (Takara; Tokyo, Japan). Primers were designed using Primer-BLAST and the sequences are presented in **Table 1**. The comparative C_t_ method was used and the relative mRNA expression level was calculated based on normalization to β-actin. All experiments were repeated three times independently.

**Table 1:** Primers used in this study.

Primer	Sequences
GM-CSF	
Forward	5’-AGATATTCGAGCAGGGTCTAC-3’
Reverse	5’-GGGATATCAGTCAGAAAGGTT-3’
SCF	
Forward	5’-GCCAGAAACTAGATCCTTTACTCCTGA-3’
Reverse	5’-CATAAATGGTTTTGTGACACTGACTCTG-3’
G-CSF	
Forward	5’-ATGGCTCAACTTTCTGCCCA-3’
Reverse	5’-AATACCCGATAGAGCCTGCA-3’
M-CSF	
Forward	5’-GAGGTGTCAGAACACTGTAG-3’
Reverse	5’-CAATCTGGCATGAAGTCTCC-3’
β-actin	
Forward	5’-GACCTGTACGCCAACACAGT-3’
Reverse	5’-CCAGGGCAGTGATCTCCTTC-3’

### Statistical analysis

Data are expressed as the mean ± standard deviation. Data in the bar and dot graphs between groups were compared using an unpaired Student’s *t*-test for normal data distribution or the Mann–Whitney non-parametric test for abnormal data distribution using GraphPad Prism (version 7.0; GraphPad Software, Inc., La Jolla, CA, USA). P< 0.05 was considered statistically significant.

## Results

### Isolation of high EPS-expressing *D. radiodurans* from the crater of Mt. Halla

**Figure 1:**
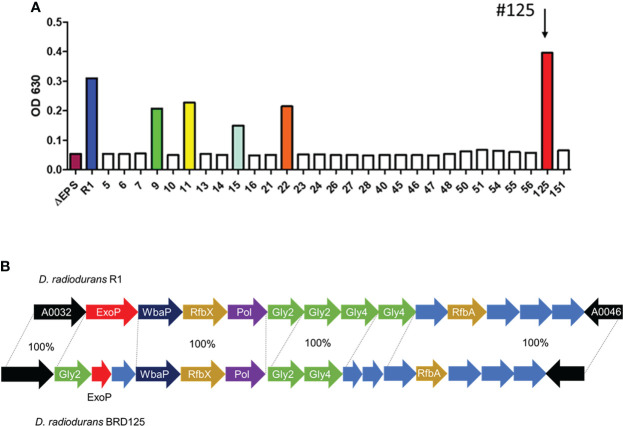
High exopolysaccharide (EPS)-producing *Deinococcus radiodurans* BRD125 isolated from the crater of Mt. Halla. **(A)** Quantification of EPS production in *Deinococcus* sp. using the anthrone assay. EPS in the culture supernatants was precipitated using 80% ethanol, and then quantified using an anthrone reaction. The bacteria culture. **(B)** Comparison of the R1 and BRD125 EPS biosynthesis gene clusters. ExoP: regulatory protein, Gly: Glycosyltransferase, WbaP: undecaprenyl-phosphate galactose phosphotransferase, RfbX: polysaccharide transporter, RfbA: glucose-1-phosphate thymidyltransferase. Blue arrow: hypothetical protein.

Soil samples were obtained from the crater of Mt. Halla, where human access has been restricted for over 20 years, with the permission of the Jeju World Natural Heritage Center (http://wnhcenter.jeju.go.kr/). We collected soil samples from the central location of the crater of Mt. Halla (33.36°N, 126.53°W), and after removing the radiation-sensitive bacteria by irradiation with 10 kGy of γ-rays, 98 radiation-resistant microorganisms were isolated including 29 *Deinococcus* sp. As deinococcal EPS showed important antioxidant and anti-radiation effects in our previous study (48), we comparatively analyzed the high expression of EPS in freshly isolated *Deinococcus* sp. in Mt. Halla. As shown in **Figure 1A**, most deinococcal isolates (n=24) did not produce EPS, which was comparable with the EPS null strain, R1ΔEPS, whereas isolates 9, 11, 15, 22, and 125 produced significant amounts of EPS and isolate 125 had the highest EPS expression, even higher than the reference strain, *D. radiodurans* R1. This strain was named BRD125. Comparative genomic analysis showed that the organization of EPS biosynthesis locus differed slightly between R1 and BRD125 (**Figure 1B**). Most polysaccharide biosynthesis genes of BRD125, such as polymerase and flipase, showed 100% identities with *D. radiodurans* R1, but sugar structure and polymerase regulation, such as ExoP and glycosyltransferase had starkly different organization and lower identities with *D. radiodurans* R1. Thus, the EPS of BRD125 might have a different structure than that of *D. radiodurans* R1. Nevertheless, the overall genomic structure of BRD125 was quite similar to that of R1 (99.7% similarity). We selected the BRD125 strain as the EPS-producing natural strain and proceeded with the following study.

### Radical scavenging activity of DeinoPol-BRD125 *in vitro*

**Figure 2:**
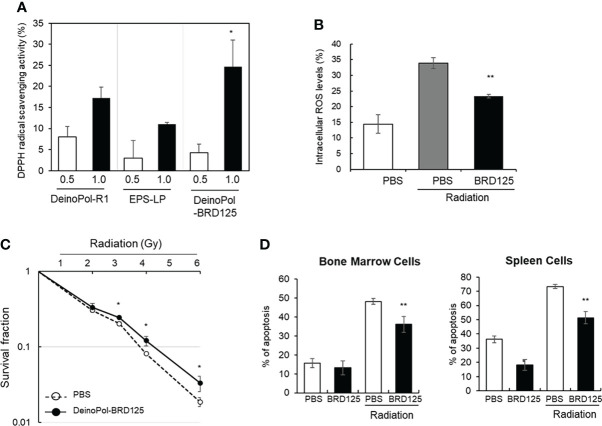
Inhibition of radical scavenging and radiation-induced cell death by DeinoPol-BRD125 *in vitro*. **(A, B)** Radiation induced radical scavenging effect by DeinoPol-BRD125. CHO-K1 cells were pre-treated with DeinoPol-R1, EPS isolated from *Lactococcus plantarum*, or DeinoPol-BRD125 (0.5 or 1.0 μg) followed by irradiation (4 Gy). DPPH scavenging activity was calculated by comparing the proportion of the amount of DPPH generated in irradiated cells with and without DeinoPol-BRD125 treatment **(A)**. *p<0.05 compared to DeinPol-R1 and EPS-LP. Intracellular ROS production in CHO-K1 cells after irradiation (4 Gy) were quantified by labeling with DCHF-DA using flow cytometry **(B)**. **(C, D)** Protection of radiation-induced cell death by DeinoPol-BRD125. CHO-K1 cells were pretreated with DeinoPol-BRD125 (50 μg/ml) for 2 h and cell survival was calculated at 7 days after irradiation using clonogenic assay **(C)**. Mouse bone marrow cells or splenocytes were pretreated with DeinoPol-BRD125 (50 μg/ml) for 2 h and the ratio of apoptosis was calculated at 1 day after irradiation (2 Gy) by staining with propidium iodide (PI) **(D)**. *p<0.05 and **p<0.001 compared to PBS-treated irradiated cells.

In our previous study, we reported that DeinoPol-R1 isolated from *D. radiodurans* R1 had radical scavenging activity and reduced UV-induced cell death in NHEK-Ad cells (48). Therefore, we confirmed the radical scavenging effect of DeinoPol-BRD125 in comparison with that of DeinoPol-R1 and EPS isolated from *Lactococcus plantarum* (EPS-LP) by measuring DPPH activities. As shown in **Figure 2A**, DeinoPol-BRD125 showed significantly higher DPPH scavenging activities than DeinoPol-R1 and EPS-LP. Subsequently, we measured the scavenging activity of intracellular ROS generated by radiation in CHO-K1 cells by pretreatment with DeinoPol-BRD125 (**Figure 2B**). Intracellular ROS levels in irradiated cells increased by 134.8 ± 14.74% compared to those of non-irradiated cells. However, when the cells were pretreated with DeinoPol-BRD125 (50 μg/ml), intracellular ROS levels were significantly reduced by 31.27 ± 2.13% compared to untreated cells.

To measure the direct physiological effect of DeinoPol-BRD125 against radiation, the survival fraction of CHO-K1 cells was examined using a clonogenic assay (**Figure 2C**). The survival fraction of CHO-K1 was decreased in a radiation dose-dependent manner, and the radiation dose at which 90% of CHO-K1 cells died was approximately 3.68 Gy. However, when CHO-K1 cells were pretreated with 50 μg/ml of DeinoPol-BRD125, the survival fraction of CHO-K1 was significantly reduced. Additionally, it was confirmed that the radiation dose at which 90% of CHO-K1 cells died increased to approximately 4.25 Gy.

### Radioprotective effect of DeinoPol-BRD125 *ex vivo*

Subsequently, we investigated the radioprotective effect of DeinoPol-BRD125 in an *ex vivo* mouse model. As ionizing radiation is likely to attack rapidly differentiating cells such as BMCs and immune cells (62), we investigated whether DeinoPol-BRD125 treatment reduces radiation-induced apoptosis in BMCs and spleen cells. BMCs and spleen cells isolated from C57BL/6 mice were plated on 96-well plates (1×10^5^ cells/well) and pretreated with DeinoPol-BRD125 followed by irradiation (2 Gy) to compare the apoptosis rate at 2 h post irradiation using *in vitro* apoptosis assay. As shown in **Figure 2D**, apoptosis of spleen cells and BMCs was significantly increased by irradiation, but it was confirmed that when these cells were pretreated with DeinoPol-BRD125 (50 μg/ml), apoptosis was reduced by 30.0 ± 1.7% and 25.1 ± 9.57% compared to untreated cells, respectively.

**Figure 3:**
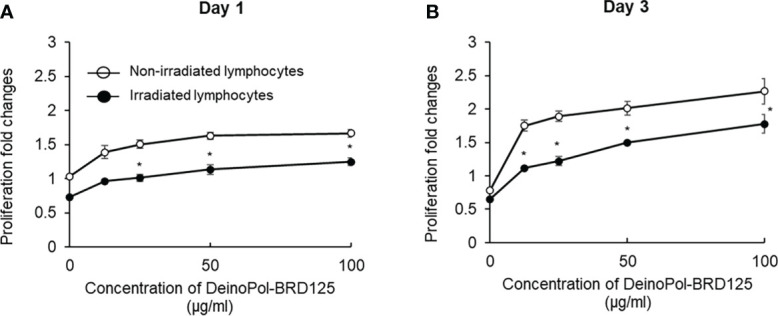
Enhancement of the proliferation of immune cells by DeinoPol-BRD125. **(A, B)** Splenic lymphocytes (3×10^5^/well) were pretreated with indicated concentration of DeinoPol-BRD125 for 2 h followed by irradiation (1 Gy). Cell proliferation was measured with bromodeoxyuridine (BrdU) proliferation assay kit at day 1**(A)** and day 3 **(B)** after irradiation. *p<0.05 compared to cells without DeinoPol-BRD125.

To investigate whether DeinoPol-BRD125 protected against radiation or enhanced the proliferation of the cells, splenic lymphocytes were irradiated at 2 Gy and the ratio of splenocyte proliferation was measured on days 1 and 3. As shown in **Figure 3**, both irradiated and non-irradiated splenic lymphocytes pre-treated with indicated concentration of DeinoPol-BRD125 exhibited increased proliferation by 33–60% and 29–69% at 1 day, and 122–188% and 70–171% at 3 days compared to untreated cells. These results suggested that DeinoPol-BRD125 had a bifunctional radioprotective effect and suppressed IR-induced apoptosis and enhanced cell proliferation.

### Radioprotective effect of DeinoPol-BRD125 *in vivo*

**Figure 4:**
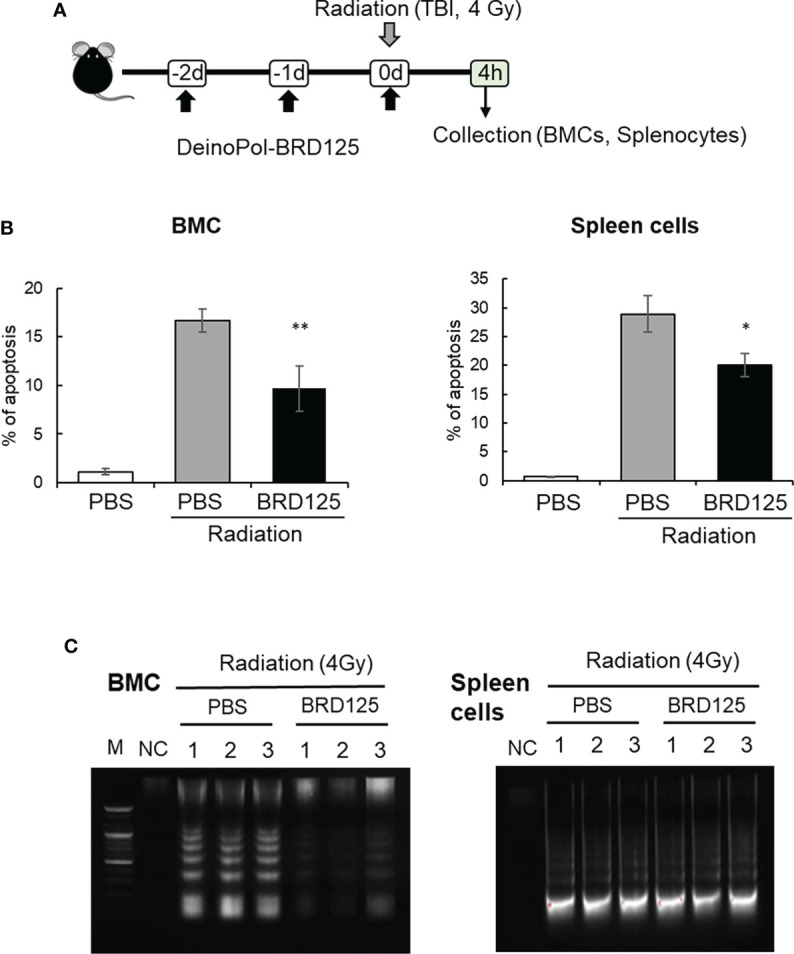
Protection of radiation-induced cell death by DeinoPol-BRD125 *in vivo*. **(A–C)** Mice (n=6) were injected intraperitoneally with DeinoPol-BRD125 (50 mg/kgBW) twice before and once after irradiation at a dose of 4 Gy. Bone marrow cells (BMCs) and spleen cells were collected at 4 h after irradiation. Schematic schedule of radiation-induced immune cell death experiment **(A)**. The ratio of apoptosis was calculated by staining BMCs (left) or spleen cells (right) with propidium iodide (PI) **(B)**. Protection of chromosomal DNA damage after irradiation by DeinoPol-BRD125 was measured by DNA fragmentation analysis. Chromosomal DNA from individual mice spleen and BMC were harvested at 4 h after irradiation and the pattern of DNA damage was visualized on 2% agarose gel. M: DNA ladder, NC: No DNA, PBS: PBS treated mice, BRD125: DeinoPol-BRD125 (00 μg/mice) treated mice **(C)**. *p<0.01 and **p<0.001 compared to PBS-injected irradiated mice.

To investigate whether the suppression of radiation-induced apoptosis by DeinoPol-BRD125 confirmed *in vitro* and *ex vitro* also occurs in the mouse *in vivo* model, mice (n = 6) orally treated with DeinoPol-BRD125 (50 mg/kg BW) were irradiated with 4 Gy (total body irradiation) (**Figure 4A**). The percentage of intracellular apoptotic cells in BMCs and spleen cells were then analyzed using PI staining and oligonucleosomal DNA fragmentation at 4 h post-irradiation. As shown in **Figure 4B**, the percentage of apoptotic cells in BMCs and spleen cells increased by 93.05 ± 0.54% and 97.43 ± 0.33% compared to those of normal control mice, respectively. However, when irradiated mice were treated with DeinoPol-BRD125 as shown in **Figure 3A**, apoptotic cells were significantly reduced by 81.36 ± 43.19% and 83.95 ± 1.94% in both BMCs and spleen cells compared to those of radiated control mice.

Another direct approach to measuring apoptotic cell death is to analyze the degradation of genomic DNA by the direct or indirect effects of radiation. Mice injected with PBS or DeinoPol-BRD125 were irradiated with 4 Gy of gamma-radiation. At 4 h after irradiation, mice were sacrificed and BMCs and spleen cells collected. Genomic DNA was isolated and visualized on 0.5% agarose gel. As shown in **Figure 4C**, smaller fragments of oligonucleosome size were visualized as a laddering pattern on an agarose gel. In BMCs and the spleen cells, genomic DNA fragmentation was markedly increased by total body irradiation at 4 Gy. Surprisingly, DeinoPol-BRD125 injection protected against the degradation of genomic DNA in BMCs but not in spleen cells. These data indicate that DeinoPol-BRD125 had a protective effect in both BMCs and spleen cells, but the molecular mechanism of protection was likely distinct in each cell type.

### Protection of hematopoietic system by DeinoPol-BRD125 in irradiated mice

**Figure 5:**
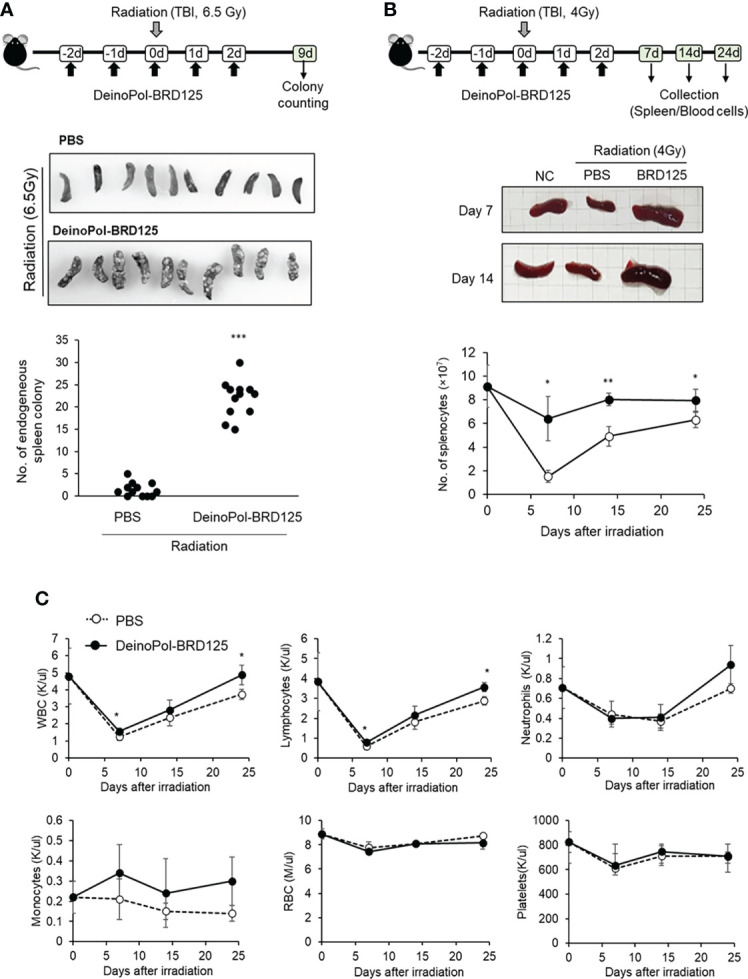
Rapid regeneration of hematopoietic stem cells by DeinoPol-BRD125. **(A)** Mice (n=12) were injected intraperitoneally twice before radiation and an additional third time after radiation with DeinoPol-BRD125 (50 mg/kgBW). Schematic schedule of endogenous spleen colony assay (top). At 9 days after 6.5 Gy radiation exposure, the colonies on the surface of the spleens were visualized (middle) and counted (bottom). **(B, C)** Mice (n=6) were injected intraperitoneally with DeinoPol-BRD125 (50 μg/BW). Schematic schedule of spleen and blood cell analysis (top). At 7, 14, and 24 days after 4 Gy radiation exposure, the spleens were visualized (middle) and total splenocytes were counted (bottom) **(B)**. At 7, 14, and 24 days after 4 Gy radiation exposure, the blood were collected in K3EDTA tubes. WBC, lymphocytes, neutrophiles, monocytes, red blood cells, and platelets were analyzed using automatic blood analyzer **(C)**. *p<0.05, **p<0.01 and ***p<0.001 compared to PBS-injected irradiated mice.

Previous studies have shown that after irradiation, rapid regeneration of multi-lineage blood cells from hematopoietic stem cells in the spleen as well as the BM can overcome ARS and ultimately improve survival (63–65). Therefore, we hypothesized that although DeinoPol-BRD125 does not protect the spleen cells from radiation, hematopoietic cells are well protected by DeinoPol-BRD125 after irradiation and migrate to aid in regeneration of spleen cells and peripheral blood cells that have been profoundly damaged by radiation. To analyze the migration and regenerative capacity of BM hematopoietic cells to the spleen, we performed endogenous CFU assays in the spleen (60, 61). This animal experiment model is summarized in **Figure 5A**. When mice were irradiated with 6.5 Gy radiation, it was confirmed that the BMCs that survived after irradiation rapidly migrated into the spleen to recruit insufficient peripheral blood cells, resulting in the formation of the colonies on the spleen surface. The number of endogenous CFUs in irradiated mice injected with DeinoPol-BRD125 was approximately 15.18-fold higher than in irradiated control mice (22.77 ± 4.85 *vs.* 1.5 ± 1.57) (**Figure 5A**). These results indicated that DeinoPol-BRD125 may play a role in protecting the stem cells of irradiated mice.

In addition, estimation of the number of spleen lymphocytes confirmed that the cell number, which had rapidly decreased after irradiation, was restored to normal when DeinoPol-BRD125 was injected (**Figure 5B**). The number of splenocytes decreased to a minimum at 7 days after irradiation, and was gradually recovered, but remained lower than that of normal mice until 24 days after irradiation. In contrast, DeinoPol-BRD125 injection alleviated the number of splenocytes, and the size of the spleen was observed to be similar to that of normal mice.

Subsequently, we analyzed the regeneration of blood cells after irradiation to investigate whether endogenous CFUs were formed by migration of blood cells. Irradiation led to a decrease in the numbers of circulating WBC, RBC, lymphocytes, neutrophils, and platelets to a minimum at 7 days, after which these cell counts partly recovered; however, irradiated mice injected with DeinoPol-BRD125 showed an increased number of these cells (**Figure 5C**). In particular, the regeneration of WBCs and lymphocytes in irradiated mice injected with DeinoPol-BRD125 were significantly increased compared to those of irradiated controls.

**Figure 6:**
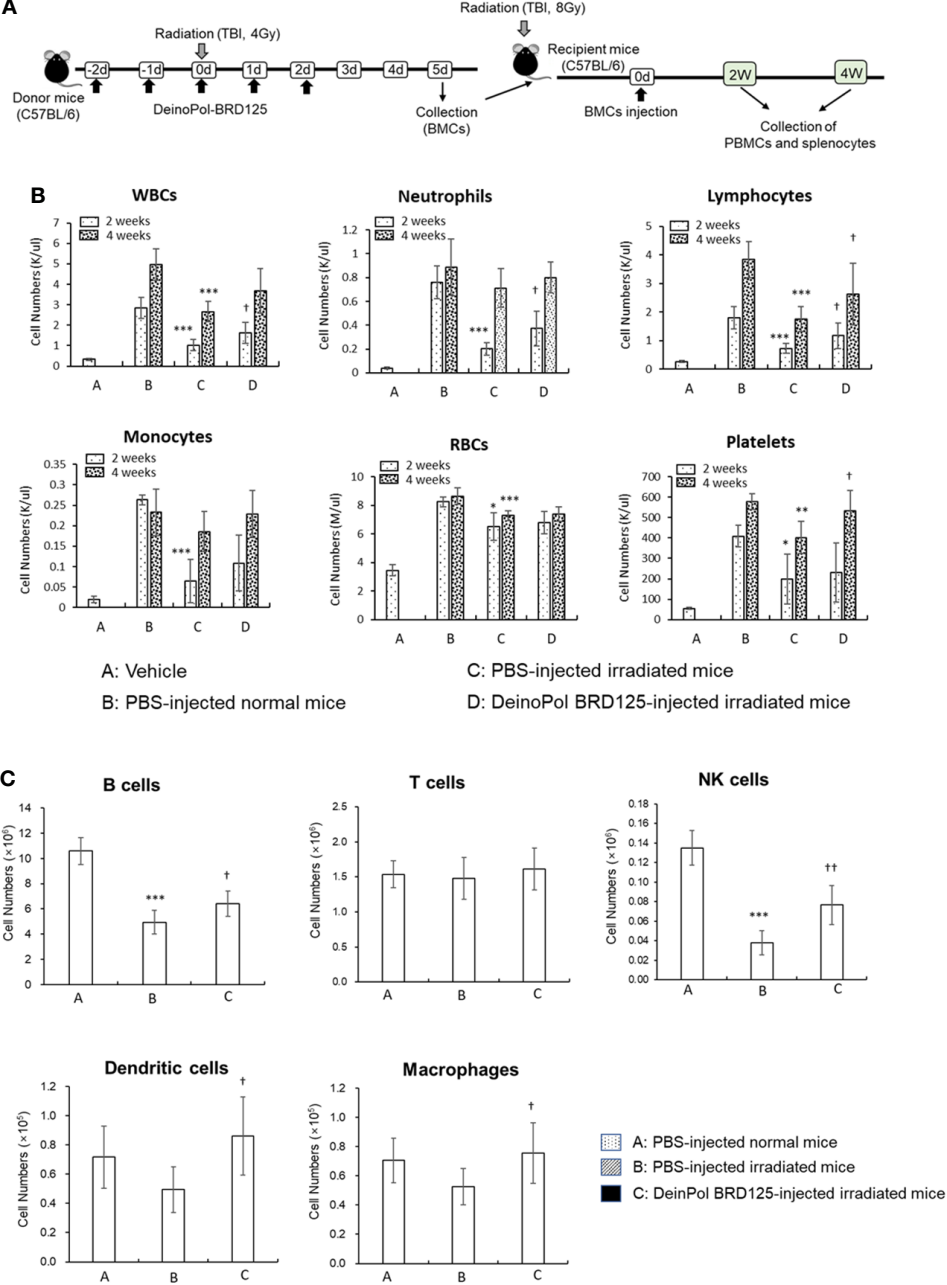
DeinPol-BRD125 enhances multilineage engraftment of irradiated hematopoietic stem cells after BM transplantation. **(A–C)** Mice were injected intraperitoneally with DeinoPol-BRD125 (50 mg/kgBW) two times before and three time after irradiation at a dose of 4 Gy. At 7 days after irradiation, bone marrow (BM) cells were injected into the tail veins of recipient mice previously administered lethal irradiation. Schematic schedule of BM transplantation experiment **(A)**. Cells in peripheral blood at 2 and 4 weeks after BM transplantation were counted by automatic blood analyzer **(B)**. Cell populations in the spleen at 2 weeks after BM transplantation were was analyzed by flow cytometry **(C)**. *p<0.05, **p<0.01, and **p<0.001 compared to recipient mice transplanted with PBS-injected normal mice. ^†^p<0.05 and ^††^p<0.01 compared to recipient mice transplanted with BM cells from PBS-injected irradiated mice.

To determine whether DeinoPol-BRD125 can protect and rescue BM hematopoietic stem cells (HSCs) from irradiation, we performed a BM transplantation experiment (**Figure 6A**). At 2 weeks after BM transplantation, all cell populations in peripheral blood of recipient mice transplanted with BM cells from PBS-injected irradiated donors were significantly decreased compared to those of recipients injected with BM cells from normal donors (**Figure 6B**). However, the recipients transplanted with BM cells from DeinoPol-BRD125-injected irradiated mice exhibited a significant increase in the number of WBCs, lymphocytes, and neutrophils compared to recipient mice transplanted with BM cells from irradiated control mice (**Figure 6B**). Furthermore, we analyzed cell populations in the spleen of recipients transplanted with BM cells. As shown in **Figure 6C**, the numbers of B cells (B220+), macrophages (MHCII+CD11c-CD11b+F4/80+), dendritic cells (MHCII+CD11C+), and natural killer cells (MHCII-CDb+NK1.1+) in splenocytes of the recipient mice transplanted with BM cells from DeinoPol-BRD125-injected irradiated mice were all increased significantly compared with those of recipients with BM cells transplanted from PBS-injected irradiated donors. These results suggest that DeinoPol-BRD125 enhances the protection of HSCs in BM cells against irradiation and increases multilineage engraftment of irradiated HSCs after BM transplantation.

**Figure 7:**
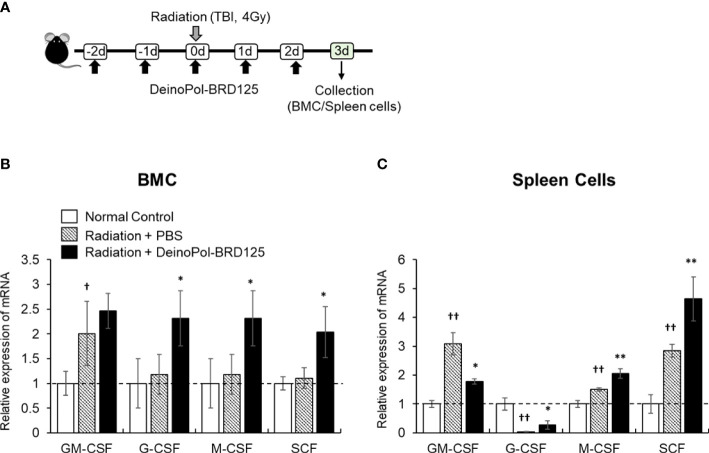
Modulation of hematopoiesis-related cytokine expression by DeinoPol-BRD125 in irradiated mice. **(A–C)** Mice (n=3) were injected intraperitoneally twice before irradiation and an additional third time after irradiation with DeinoPol-BRD125 (50 mg/kgBW). Bone marrow cells (BMC) and spleen cells were collected at 3 days after irradiation. Schematic schedule of hematopoietic stem cell cytokine expression experiment **(A)**. mRNA expressions of hematopoietic-related cytokine (GM-CSF, G-CSF, M-CSF, SCF) in BMC **(B)** and spleen **(C)** were quantified using real time PCR. ^†^p<0.05 and ^††^p<0.01, compared to normal control; *p<0.05 and **p<0.01 compared to PBS-injected irradiated mice.

Finally, mRNA expression of hematopoietic-related cytokines such as GM-CSF, G-CSF, M-CSF and SCF was analyzed in the spleen cells and BMCs of irradiated mice. This animal experiment model is summarized in **Figure 7A**. In BM, all hematopoietic-related cytokines except GM-CSF increased rapidly after injection of DeinoPol-BRD125 (**Figure 7B**). In spleen cells, GM-CSF, M-CSF, SCF were significantly increased, and G-CSF were significantly decreased in the spleen of irradiated control mice (**Figure 7C**). However, when mice were injected with DeinoPol-BRD125, G-CSF and M-CSF were more expressed in the spleen than in the irradiated control group while GM-CSF expression was down-regulated. As the spleen is known to undergo extramedullary hematopoiesis in hematopoietic emergencies, the radioprotective effect of DeinoPol-BRD125 might result from the rapid recovery of hematopoiesis to provide the necessary protection against radiation by enabling rapid regeneration of BMCs and blood cells.

## Discussion

Whole-body exposure to radiation causes disorders in various organs and tissues, resulting in complicated clinical developments, collectively termed ARS. ARS is classically divided into three subsyndromes: hematopoietic, gastrointestinal, and neurovascular syndromes as it especially depletes the immature parenchymal stem cells of those tissues (26–29, 66). There are several theories explaining the pathophysiological mechanisms of ARS, such as radiation-induced mass death of parenchymal cells and indirect radiation effects on cells and tissues (7, 67). Among them, hematopoietic ARS is the most common, occurring at an irradiation dose of 0.7 to 10 Gy (30, 67). However, there are currently no effective and safe approaches for treating hematopoietic ARS. We reported here that bacterial EPSs isolated from the extremely radiation resistant bacteria *D. radiodurans* conferred a strong protective effect against ARS. Although there are several drugs to minimize radiation-induced damage, their use has been very limited owing to severe side effects such as nausea, vomiting, and hypotension (19, 20). In contrast, DeinoPol-BRD125 is a non-toxic polysaccharide that can minimize RT-induced damage by rapidly restoring hematopoiesis.

Radiation can cause long-term BM damage owing to defects in HSC self-renewal capacity and HSC senescence (64, 68). As an excessive amount of various free radicals generated indirectly by radiation is an important cause of tissue and organ damage, considerable attention has been paid to the development of radioprotectors for scavenging radiation-induced free radicals (7, 9, 69). However, simply removing ROS is insufficient to completely prevent damage. This is because the tissue damage caused by radiation exposure is rather complex, with various pathophysiological effects occurring simultaneously (7, 9, 11). As an ideal method for overcoming radiation injury, many oncologists and radiation biologists have proposed to accelerate the recovery of blood cells while simultaneously protecting HSCs from radiation (10, 63). Chemical agents such as amifostine, various cytokines, and numerous microbial components such as bacterial lipopolysaccharide and *Mycobacterium bovis* strain BCG have been studied as radioprotective and radiomitigator agents with competitive scavenging of free radicals or enhancement of hematopoietic and immune functions (10, 69–72). However, because these agents have severe toxicity or side-effects *in vivo*, it is difficult to use them in patients who have received high doses of radiation. For this reason, an ideal radioprotector is considered to be one that confers a high degree of protection to normal tissues and, most importantly, is nontoxic (7, 67, 73). In the present study, we showed that DeinoPol-BRD125 exhibited no significant toxicities *in vitro*. Although DeinoPol-BRD125 is less toxic compared to available radioprotective drugs, comparative evaluation studies of the radioprotective efficacy will be needed in the future.

Microbial EPSs are high molecular weight, non-toxic polysaccharide compounds secreted in the extracellular space. They have diverse biological functions, such as environmental protection, surface adherence, biofilm formation, and intercellular interactions (42, 44, 74–76). Purified EPSs are biocompatible and ecofriendly high-value biomolecules, and serve as antimicrobials, anticancer drugs, antioxidants, drug delivery systems, and chemical sensors (77–80). For example, the EPS produced by *Lactobacillus kefiranofaciens* DN1 exhibited bactericidal and bacteriostatic activities against many food-borne pathogens by directly disrupting the structure of their cell wall architecture (35, 80). The EPS of the probiotic stain *Bifidobacterium breve* lw01 isolated from infant fecal samples was shown to possess an anticancer activity *in vitro* (81). In addition, our previous studies indicated that *D. radiodurans* produces several extracellular biomolecules that have antioxidant, anti-biofilm, and anti-allergic effects (48, 55). In this study, we investigated the biological effects of DeinoPol-BRD125 from newly isolated *D. radiodurans* BRD125 strain from the crater of Mt. Halla, which had 96% genomic homology with the reference strain, *D. radiodurans* R1. Although we have not biochemically compared the structural differences of the two DeinoPols (BRD125 *vs.* R1) in this study, BRD125 showed markedly improved radical scavenging, cell protection, and hematopoiesis recovery effects. Thus, DeinoPol-BRD125 likely has a different or modified polysaccharide structure compared to DeinoPol-R1.

Generally, HSCs are highly sensitive to radiation exposure; therefore, protecting these cells from excessive free radicals is very important (7, 29, 67). We also found that whole body radiation (4 or 6.5 Gy) drastically reduced cell numbers in the spleen, BM, and peripheral blood. In addition, we confirmed that hematopoiesis slowly recovered in the BM and returned to normal after approximately 24 days. In the case of urgent hematopoiesis necessity such as pregnancy, infection, myeloablation, and radiation exposure, HSCs migrate from the BM to the spleen, and hematopoiesis subsequently occurs in the spleen (82–84). Our study showed that the expression of hematopoiesis-related cytokines after irradiation markedly increased in the spleen but not in the BM, suggesting that hematopoiesis within the spleen can be just as important as that in the BM. DeinoPol-BRD125 administration increased the number of splenic CFUs in irradiated mice, and the expression of GM-CSF, G-CSF, M-CSF, and SCF was also increased in both the BM and spleen of irradiated mice. Thus, our findings strongly suggested that DeinoPol-BRD125 protects the hematopoietic system and promotes hematopoiesis in the spleen as well as in the BM to overcome ARS.

In the field of radiation safety research, interest in the development of ideal radioprotective and mitigating agents is continuously increasing, with a focus on developing substances that inhibit apoptosis and increase regeneration while being nontoxic. To the best of our knowledge, ours is the first study to use a natural nontoxic microbial EPS component as an ideal radioprotector and mitigator. In addition, DeinoPol-BRD125 can be proposed as both a radioprotector drug as well as a “functional” food for radiotherapy patients suffering from long-term ARS.

## Data availability statement

The datasets presented in this study can be found in online repositories. The names of the repository/repositories and accession number(s) can be found in the article/Supplementary Material.

## Ethics statement

This study was performed in strict accordance with the recommendations of the Guide for the Care and Use of Laboratory Animals of the National Institutes of Health. All animal experiments were approved by the Committee on the Use and Care of Animals at the Korea Atomic Energy Research Institute (KAERI; approval no. KAERI-IACUC-2021-003) and performed according to accepted veterinary standards set by the KAERI animal care center. Mice were euthanized by CO2 inhalation, as specified by the KAERI Institutional Animal Care and Use Committee guidelines.

## Author contributions

HRP, KBA, and HSS conceptualized the study, HRP, JHL, and HJJ performed the experiments and analyzed the data. HRP and HSS wrote the manuscript. HSS supervised the work. SL, KBA, and HSS were responsible for funding acquisition. All authors contributed to the article and approved the submitted version.

## Funding

This work was supported in part by the Internal R&D program of KAERI (523210) funded by Ministry of Science and ICT (MSIT) and the National Research Foundation of Korea grants 2017M2A2A6A02020925, NRF-2018K2A206023828, and NRF-2020M2A206023828 to HSS and NRF-2019M2D2A2060217 to KBA.

## Acknowledgments

We acknowledge the Jeju World Natural Heritage Center and Jeju Provincial Government for their support in isolating the *Deinococcus* strains for this study.

## Conflict of interest

The authors declare that the research was conducted in the absence of any commercial or financial relationships that could be construed as a potential conflict of interest.

## Publisher’s note

All claims expressed in this article are solely those of the authors and do not necessarily represent those of their affiliated organizations, or those of the publisher, the editors and the reviewers. Any product that may be evaluated in this article, or claim that may be made by its manufacturer, is not guaranteed or endorsed by the publisher.
