# Supplementary figures and images for: Radioprotection of deinococcal exopolysaccharide BRD125 by regenerating hematopoietic stem cells
Source: Front Oncol. 2022 Sep 26;12:898185. doi: 10.3389/fonc.2022.898185 (PMC9549790; doi:10.3389/fonc.2022.898185)

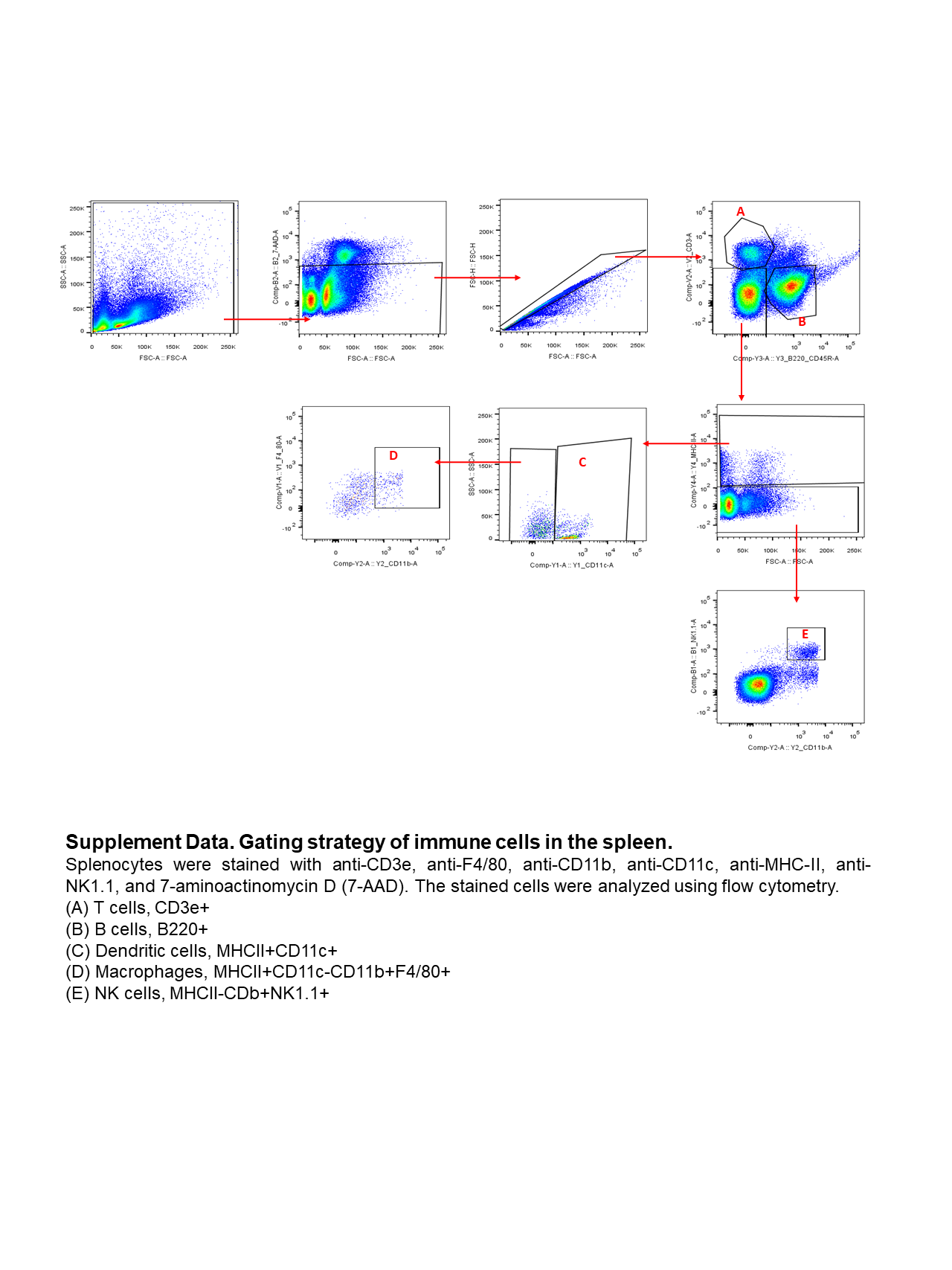

Supplement: Supplementary file 1 [file Image_1.tif]
